# Estrogen Receptor β Activation Impairs Prostatic Regeneration by Inducing Apoptosis in Murine and Human Stem/Progenitor Enriched Cell Populations

**DOI:** 10.1371/journal.pone.0040732

**Published:** 2012-07-10

**Authors:** Shirin Hussain, Mitchell G. Lawrence, Renea A. Taylor, Camden Yeung-Wah Lo, A. P. C. BioResource, Mark Frydenberg, Stuart J. Ellem, Luc Furic, Gail P. Risbridger

**Affiliations:** 1 Prostate & Breast Cancer Research Program, Department of Anatomy & Developmental Biology, Monash University, Clayton, Victoria, Australia; 2 Monash Micro Imaging, Monash Health Translation Precinct, Clayton, Victoria, Australia; 3 Australian Prostate Cancer BioResource, Victorian Node, Monash University, Clayton, Victoria, Australia; 4 Department of Surgery, Faculty of Medicine, Monash University, Clayton, Victoria, Australia; The University of Texas M.D Anderson Cancer Center, United States of America

## Abstract

Androgen depletion is the primary treatment for prostate disease; however, it fails to target residual castrate-resistant cells that are regenerative and cells of origin of prostate cancer. Estrogens, like androgens, regulate survival in prostatic cells, and the goal of this study was to determine the advantages of selective activation of estrogen receptor β (ERβ) to induce cell death in stem cells that are castrate-resistant. Here we show two cycles of short-term ERβ agonist (8β-VE2) administration this treatment impairs regeneration, causing cystic atrophy that correlates with sustained depletion of p63+ basal cells. Furthermore, agonist treatment attenuates clonogenicity and self-renewal of murine prostatic stem/progenitor cells and depletes both murine (Lin^−^Sca1^+^CD49f^hi^) and human (CD49f^hi^Trop2^hi^) prostatic basal cells. Finally, we demonstrate the combined added benefits of selective stimulation of ERβ, including the induction of cell death in quiescent post-castration tissues. Subsequent to castration ERβ-induces further apoptosis in basal, luminal and intermediate cells. Our results reveal a novel benefit of ERβ activation for prostate disease and suggest that combining selective activation of ERβ with androgen-deprivation may be a feasible strategy to target stem cells implicated in the origin of prostatic disease.

## Introduction

Androgen deprivation therapy (ADT) has been the standard of care for prostatic disease since 1941, when a landmark publication by Huggins and Hodges showed significant clinical benefit to prostate cancer patients treated with either surgical or chemical castration [1]. In this setting, estrogens were administered to reduce hypothalamic pituitary stimulation of LH/FSH production and thus suppress androgen synthesis. Despite the therapeutic effectiveness of this approach, estrogens had significant cardiovascular and thrombotic side-effects which led to the decline in their use [2]. More recently, a second estrogen receptor, estrogen receptor β (ERβ), was discovered in the human prostate gland [3]. This added further complexity to understanding the mechanism of action of estrogens, and prompted us to re-evaluate the potential for estrogens to be used therapeutically for prostate disease [3].

Studies using ERβ knockout mouse models have been conflicting and controversial. The variable prostatic phenotypes observed in these studies to date [4] have prevented elucidation of the functional roles or downstream targets of estrogen acting via ERβ, although anti-proliferative activity was initially postulated [5]. As an alternate approach, selective ER agonists (α or β) can be used to define the beneficial actions of estrogen via ERβ that contrast with those mediated by ERα. In a series of studies using 8β-VE2, a selective ER agonist with <75-fold selectivity for ERβ over ERα [6], we showed a direct local action via ERβ that is distinct to ERα induced activity. In the first study, we used tissue recombinants to show the agonist abrogated prostatic hyperplasia of ArKO (Aromatase knockout) mice, which lack endogenous estrogens, by inhibiting cell proliferation and stimulating apoptosis [7]. Subsequently, we showed anti-proliferative and pro-apoptotic responses to ERβ agonist in both a testosterone replete and deplete milieu [8]. Thus, in contrast to castration, ERβ-induced apoptosis occurs independently of testosterone manipulation. ERβ-induced apoptosis is also mechanistically different, using the TNFα extrinsic apoptotic signaling pathway rather than the intrinsic pathway activated by castration. Dependency on both ERβ and TNFα was proven using siRNA and knockout mouse models [8].

A further difference to castration, and a notable benefit of ERβ activation, is the ability to induce apoptosis in normal prostatic basal cells, human prostate cancer xenografts and castrate-resistant prostate cancer cell lines [8]. The importance of targeting castrate-resistant cells is two-fold: firstly, in prostate cancer, it is castrate-resistant cells that evade therapy and permit disease progression [9], [10], [11], and secondly, the basal cells are believed to house the regenerating stem cell populations in the normal prostate, which are also proven cells of origin of prostate cancer [12]. Evidence to support this was reported over 20 years ago when Isaacs and colleagues showed that repeated cycles of androgen deprivation and restoration resulted in continuous prostatic regeneration in the rodent because of residual stem cell activity in the basal cells [13]. More recently, in 2010, Witte and co-workers identified an important sub-fraction of murine (Lin^−^Sca-1^+^CD49f^+^; LSCs) and human (CD49f^hi^Trop2^hi^) basal cells that contain the castrate-resistant stem cells that are also a target for prostate cancer initiation [12], [14].

Our previous data suggested the response to ERβ agonist is markedly different to castration and unlike the classic studies by Isaacs [13], the impact of ERβ stimulation on regeneration over repeated cycles is unknown. Thus, the goal of this study was to determine whether stimulation of ERβ would enhance the effects of castration by inducing cell death in castrate-resistant cells via an androgen-independent mechanism. We hypothesized this would occur via targeting of a unique subset of castrate-resistant cells that fail to respond to ADT. Here we show that 8β-VE2 initiates apoptosis in basal cells that withstand castration, permanently impairs prostate regeneration *in vitro* and *in vivo,* and reduces the number of stem-enriched LSCs. Collectively, the action and cellular targets of 8β-VE2 endorse the potential of this compound as a means to target castrate-resistant cells in the prostate.

## Materials and Methods

### Animals

C57BL6/J wild type mice were housed at Monash University in accordance with the Australian National Health and Medical Research Council Guidelines for the Care and Use of Laboratory Animals.

### Ethics Statement

Studies were conducted with approval from the Monash School of Biomedical Science Animal Ethics Committee and the Monash Medical Center Animal Ethics committee (Ethics Approval numbers SOBSA-A-2010-36, MMCA-2009-61 and MMCA-2008-09). Human epithelial cell lines in this study were established from samples acquired with informed, written consent from patients and approval from the Cabrini Human Research Ethics (03-14-04-08) and the Monash University Human Research Ethics Committee (Ethics Approval number 2004/145).

### Treatments

The ERβ specific agonist 8-vinylestra-1,3,5 (10)-triene-3,17β-diol (8β-VE2) was provided by Dr. Fritzemeier from Bayer-Schering Pharma AG, Berlin, Germany. The selectivity of this drug has been described previously [6]. Testosterone replacement, castration and 8β-VE2 treatments were conducted as previously described (10).

### Human Primary Cell Culture

Specimens were obtained from 3 patients undergoing radical prostatectomy with informed consent. Benign regions of prostate tissue were dissected by a Urological Pathologist and primary epithelial cell cultures were established in Keratinocyte Serum-Free Medium supplemented with 50 ng/ml bovine pituitary extract (BPE), 5 ng/ml epidermal growth factor (EGF; Gibco, Invitrogen, Blackburn, VIC, Australia) and antibiotics (100 IU/ml penicillin and 10 µg/ml streptomycin; Invitrogen, Blackburn, VIC, Australia) at 37°C in a humidified atmosphere of 5% CO_2_.

### Spheroid Assay

The spheroid assay was performed using previously reported methods with minor modifications [15], [16]. Briefly, prostates were dissected from 8 week old C57BL/6 mice, minced, and digested with 1 mg/mL collagenase I and then 0.05% trypsin/EDTA. Cell suspensions were passed through 18 and 21 gauge syringes and 40 µm cell strainers and then incubated with Red Blood Cell Lysis Buffer (Sigma-Aldrich, Castle Hill, NSW, Australia). To generate primary spheroids, 2×10^4^ unfractionated prostate cells were seeded per 12 well in a 2∶3 mixture of PrEGM medium (Lonza, Mt. Waverley, VIC, Australia) to Matrigel (BD, North Ryde, NSW, Australia). The cells were treated with 6 µM 8β-VE2, or ethanol vehicle control, for 7 days with daily media exchanges. To passage colonies, Matrigel was digested with dispase (Sigma-Aldrich, Castle Hill, NSW, Australia) and spheroids disaggregated with 0.05% trypsin EDTA, passed through syringes, 40 µm cell strainers and reseeded in fresh PrEGM and Matrigel. Colonies were imaged using a Leica DM IL microscope fitted with a DFC 425C digital camera. The maximum diameter of colonies was measured using Image J software.

### Flow Cytometry

Flow cytometry was performed based on previously described protocols [12], [16]. Briefly, prostate lobes were collected from mice and then dissociated into single cell suspensions by mincing and collagenase digestion as previously described [17]. Red blood cells were lysed using RBC lysis buffer (Sigma-Aldrich, Castle Hill, NSW, Australia) and single cells were then re-suspended in DMEM with 10% FCS. Mouse cells were stained with anti-annexin V-pacific blue (Invitrogen, Blackburn, VIC, Australia) and propidium iodide (Sigma-Aldrich, Castle Hill, NSW, Australia) for 15 minutes at 4°C to analyse apoptosis. To identify the stem/progenitor population, mouse cells were stained with anti-CD49f-PE, anti-Sca-1-APC, anti-Ter119-FITC, anti-CD45-FITC and anti-CD31-FITC antibodies (all eBioscience, San Diego, CA, USA) for 20 minutes at 4°C. Primary human prostate epithelial cells were dislodged from flasks using TrypLE Select (Invitrogen, Blackburn, VIC, Australia), resuspended in DMEM with 10% FCS, and stained with anti-CD49f-PE (eBioscience, San Diego, CA, USA) and anti-Trop2-APC (R&D Systems, Emeryville, CA, USA) antibodies. After staining, cells were washed to remove excess antibody, and analysed on a BD LSRII flow cytometer.

### Immunohistochemistry

Immunohistochemical staining was performed as previously described [7]. ERβ, (Leica Microsystems, North Ryde, NSW, Australia), p63 (Santa Cruz Biotechnology, Santa Cruz, CA, USA), cytokeratin 18 (CK18; Progen Biotechnik, Heidelberg, Germany) and high molecular weight cytokeratin (HMWCK; Abcam, Cambridge, UK) primary antibodies were used with previously described protocols [18], [19], [20]. For Periodic Acid Schiff (PAS), formalin fixed sections were dewaxed and hydrated in water, and then oxidised in 0.5% periodic acid (Amber Scientific, Midvale, WA, Australia) for 5 minutes. Slides were rinsed in water, and then placed in Schiff’s reagent (Amber Scientific, Midvale, WA, Australia) for 15 minutes, before being ‘pinked’ in warm water. A haematoxylin counterstain is then applied before slides and dehydrated and cover-slipped. Apoptosis was detected using ApopTag® Plus Peroxidase In situ Apoptosis detection kit (Chemicon, Temecula, CA, USA) according to the manufacturer’s instructions. Quantitation of immunolocalization was conducted by uniform systematic random sampling of at least 6 sections per tissue per treatment, using the new-CAST component (version 2.14; Visiopharm, Hørsholm, Denmark) of Visiopharm Integrator System (version 2.16.1.0; Visiopharm) as previously described [7], [21].

### Dual Immunofluorescent Staining of Paraffin Sections

Immunofluoroscent staining was performed as previously described [7]. Briefly, sections were incubated with the CK18 primary antibody (1∶50) at room temperature for 1 hour. After washing in 0.05% Tween in TBS, sections were stained with anti-mouse IgG1-Alexafluor 555 (1∶500 dilution; Invitrogen, Blackburn, VIC, Australia). Sections were then stained for CKHMW (1∶300) at room temperature for 1 hour followed by anti-rabbit-Alexa Fluor 488 (1∶500 dilution; Invitrogen, Blackburn, VIC, Australia) and then mounted in Vectashield-DAPI (Vector Laboratories, Burlingame, CA, USA). Images were captured on an Olympus FSX100 microscope using a 10x 0.4NA objective in a 5×5 field-of-view tiling process to sample as large an area as possible. DAPI, Alexa Fluor 488 and Alexa Fluor 568 signals were sequentially detected using U-MNUA2, U-MWIBA3 and U-MWIG3 Olympus filter sets respectively, and a metal halide fluorescence lamp. Images were analyzed using ImageJ (National Institutes of Health) where cells were counted by their DAPI signal and categorized by their Alexa Fluor 488 and Alexa Fluor-568 signal.

#### Stereology

All assessments were performed using a Bioprecision2 Microscope Stage (Ludl, NY), 99A400 Focus drive (Ludl), MAC5000 Controller (Ludl), and ND-281 Encoder (Heidenhain, IL) coupled to an Olympus BX-51 microscope (Olympus, Tokyo, Japan). The images were captured using a PixeLink PL-623C digital camera (PixeLink, Ottawa, ON, Canada) coupled to a computer. The newCAST component (version 2.14; Visiopharm, Hørsholm, Denmark) of the Visiopharm Integrator System (version 2.16.1.0; Visiopharm) was used to generate a set of counting frames and a point grid (grid properties were assessed individually for each marker). Uniform systematic random sampling was then used to quantitate the number of positive cells on immunohistochemical stained sections, as previously described [20], [22]. In brief, a minimum of 6 sections per animal were examined at ×40 magnification; they were mapped to define tissue boundaries and were sampled at predetermined intervals along the x- and y- axes using a single point grid-counting frame. The number of positive cells was counted for each gland with at least 500 cells counted per section. The final data were expressed as the number of positive cells per unit area or per cell type.

### Statistics

Analyses were conducted using Prism 5.0d software (GraphPad Software Inc.). Data that were normally distributed were analyzed using *t* tests or ANOVA; alternatively the non-parametric Mann Whitney test was used. Significance was accepted at *P*<0.05. Data are expressed as mean ± SEM, unless otherwise stated.

## Results

### Two Cycles of Short Term ERβ Agonist Administration Impair Regenerative Capacity

**Figure 1:**
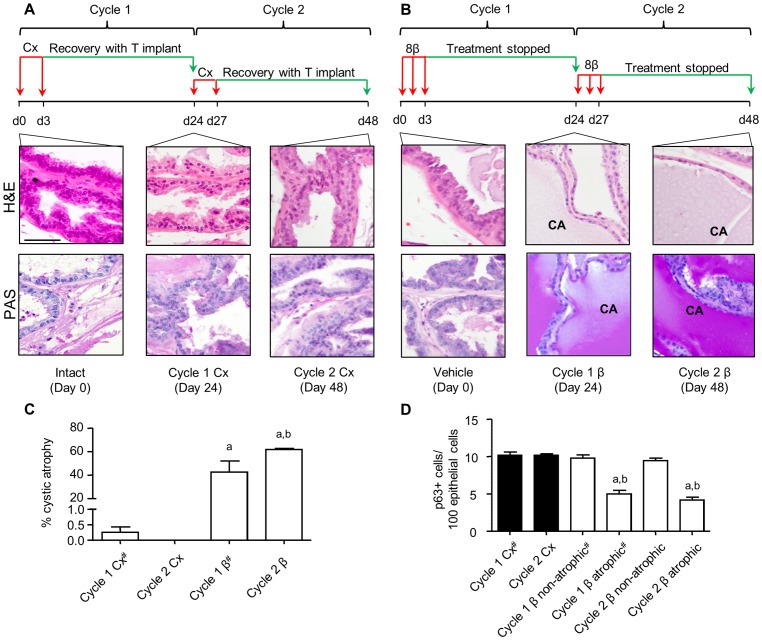
Two cycles of short term ERβ agonist administration impair ventral prostate regenerative capacity. (A) Schematic of cyclic castration (Cx) and testosterone replacement over the course of 2 cycles and representative micrographs showing haemotoxylin and eosin (H&E) staining for histology of intact (day 0), 1 cycle (day 24) and 2 cycle (day 48) Cx-recovery tissue. Second row of images in this panel shows lack of mucinous Periodic Acid-Schiff’s (PAS) staining in castrate-recovery (Cx) tissue (B) Schematic of cyclic 8β-VE2 treatment over the course of 2 cycles along with representative micrographs showing H&E staining for histology of vehicle treated (day 0), 1 cycle (day 24) and 2 cycle (day 48) 8β-VE2-recovery tissue. CA denotes areas of cystic atrophy. Second row of micrographs depicts mucinous PAS positive staining in the secretions within the lumens of ERβ agonist treated recovery tissue. (Scale bar for all images is 20 µm). (C) Stereological analysis of percent atrophy (n = 4–5 animals/group, One-Way ANOVA with Tukey’s post-hoc analysis, ^a^P<0.001 vs. corresponding castrate tissue, ^b^P<0.05 vs. cycle 1 tissue 8β-VE2). (D) Frequency of p63 positive cells per 100 luminal cells (n = 7 animals/group, One-Way ANOVA with Tukey’s post-hoc analysis, ^a^P<0.001 vs. corresponding castrate tissue, ^b^P<0.001 vs. non-atrophic region). # indicates values from (8).

Although the adult prostate is growth quiescent, repeated cycles of androgen deprivation and replacement promote continual prostatic regeneration in the rodent [13]. Previously, we showed that a single transient exposure to ERβ agonist perturbed prostatic epithelial regeneration of the ventral prostate, 21 days later, even if androgen levels were supplemented by testosterone implants [8]. To test if perturbation of regeneration persisted with a second cycle of agonist, a similar protocol to that used by Isaacs was adopted [13]. Adult WT mice were treated with 8β-VE2 or castrated for 3 days, then allowed to recover for 21 days, either with T implants in the case of castrate (Fig. 1A), or with cessation of treatment for ERβ agonist (Fig. 1B); this regimen was performed over two cycles of treatment and recovery. After one or two cycles of treatment, castrate-recovery tissues were morphologically indistinguishable from intact mouse prostate, demonstrating the typical regenerative potential of the gland (Fig. 1A). In contrast, ERβ agonist treated tissues were unable to regenerate normally and prominent areas of cystic atrophy were observed, identified by a flattened epithelium and distension of the lumen due to an increase in mucinous PAS-rich secretions (Fig. 1B). This phenotype observed after one cycle was augmented after cycle two. No lobe differences were noted and there was no significant weight change in any prostate lobes after 3 days or 2 cycles of treatment (data not shown). When quantitated using unbiased stereology, analysis of the entire ventral prostate gland showed cystic atrophy in 42.5% of agonist-treated tissues after 1 cycle of treatment, significantly increasing to 61.7% following the second cycle (Fig. 1C). Both values were significantly different to castrate-recovery tissues where no cystic atrophy was detectable (Fig. 1C). This loss of regenerative activity observed following 8β-VE2 treatment suggests that, as opposed to castration, 8β-VE2 treatment impairs the activity of prostate cells with regenerative potential.

Castration causes atrophy of the prostate with apoptosis mainly confined to the luminal cell layer, whereas the basal cells are castrate-resistant [8], [13]; selective activation of ERβ causes apoptosis in luminal and basal cells as previously described [8]. To investigate the underlying differences in the cellular mechanisms of castration compared to ERβ agonist action, we quantified the changes in the different prostatic cell populations. Basal cells were quantified by nuclear position close to the basement membrane whereas luminal cells were identified by their tall columnar appearance and nuclear position. Using stereology, the basal epithelial cell layer represents a small proportion (∼6%) of the normal epithelium and the bulk is mainly luminal cells (∼79%); the stroma is ∼15% of tissue (Fig. S1A). All of these epithelial cellular sub-populations express ERβ [23], [24], [25], [26], [27].

Previously we reported apoptosis as absolute levels in the entire tissue [8]. Given the significant differences in the proportion of cell types (Figure S1A), we repeated our analyses to express the levels of apoptosis per cell type including basal or luminal. Figure S1B shows that luminal cell apoptosis in castrate and 8β-VE2 agonist treated tissue (8 and 7% respectively) is significantly higher than control. In the minor basal cell population, no detectable apoptosis is observed after castration whereas up to ∼15% of basal cells undergo apoptosis after 3 days of 8β-VE2 agonist treatment (Fig. S1C).

Altogether, the effect of agonist on basal cells prompted the evaluation of p63^+^ basal cell frequency in the cyclic study presented above where regeneration is impaired. Using immunohistochemistry, we identified and quantitated p63^+^ basal cells in cyclic castrate and agonist treated tissues using unbiased stereology and showed that castration does not affect basal p63^+^ cell number, even after two cycles of androgen deprivation and restoration (Fig. 1D). The areas of cystic atrophy increase with each cycle of 8β-VE2 treatment and there is a persistent depletion of p63^+^ basal cells after each cycle of treatment localized to these areas. These results concur with evidence that p63^+^ basal cells are necessary for normal regeneration of the prostatic epithelium and that different to castration, these cells are 8β-VE2 sensitive.

### ERβ Attenuates the Clonogenicity and Self-renewal of Murine Prostatic Stem Cells

**Figure 2:**
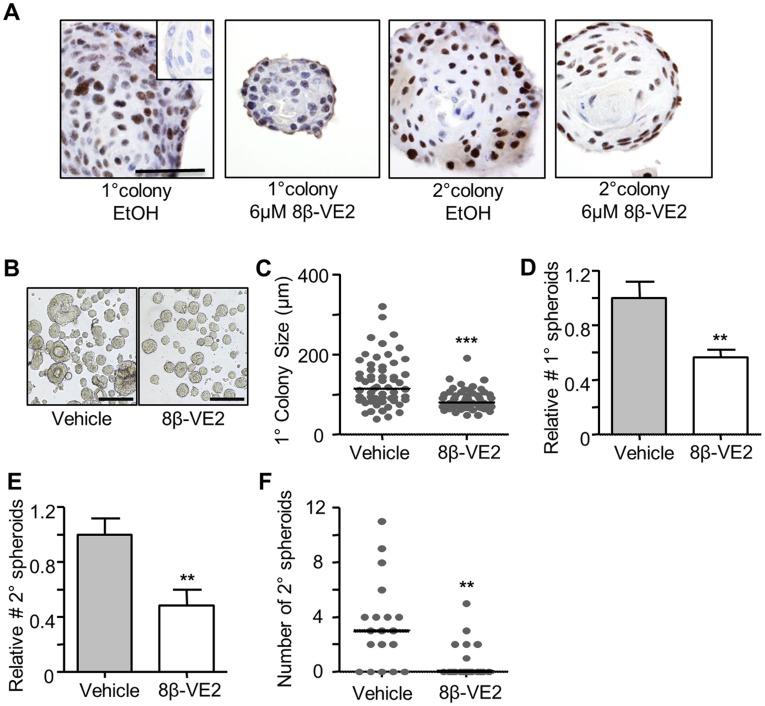
ERβ attenuates the clonogenicity and self-renewal of murine prostatic stem cells. (**A**) Representative micrographs of ERβ immunohistochemistry of primary and secondary colonies extracted from Matrigel (Inset; isotype negative control, Scale = 50 µm) (**B–D**) Formation of primary spheroids from mouse prostate cells cultured in Matrigel for 7 days with vehicle or 6 µM 8β-VE2. (**B**) Images of primary colonies extracted from Matrigel on day 7 (Scale = 250 µm). (**C**) The median size (µm) of primary colonies (***P<0.0001, Mann Whitney test, n = 60). (**D**) Relative number of primary spheroids formed with vehicle or 8β-VE2 treatment (n = 7 mice **P<0.01, T test). (**E**) Relative number of secondary spheroids arising from pooled primary colonies replated for 7 days without treatment (n = 7 mice, **P<0.01, T test). (**F**) Median number of secondary spheroids derived from individually passaged primary spheres (**P<0.01, Mann Whitney test, n = 19).

Prostate basal cells contain a sub-population of regenerative stem/progenitor cells [28], [29]. To determine if 8β-VE2 abrogates prostatic renewal by targeting stem/progenitor cells, the clonogenicity and proliferation of these cells was examined using an *in vitro* spheroid assay that selects for multi-potent, self-renewing prostate epithelial cells that form clonally-derived colonies [15]. Importantly, ERβ is expression is maintained in both primary and secondary spheroids in this assay, regardless of 8β-VE2 treatment (Fig. 2A). However, as shown in Figure 2B, and quantitated in Figure 2C, 8β-VE2 treatment significantly reduced the median size of spheroids by 30.0% and the relative number of spheroids that formed from unfractionated mouse prostate cells by 43.5% (Fig. 2D). To determine whether this initial 8β-VE2 treatment could inhibit subsequent self-renewal, primary colonies were pooled and re-cultured without agonist. As shown in Figure 2E, there was a 51.8% decrease in the number of secondary spheroids that formed from cells treated with 8β-VE2, demonstrating that the agonist abrogated the self-renewal ability of stem/progenitor cells in the primary spheroids. To verify this result, individual primary colonies were passaged and allowed to form clonal secondary colonies without treatment. Once again, colonies that were initially treated with 8β-VE2 formed significantly fewer secondary spheroids than the vehicle control (Fig. 2F). Secondary colony formation was lower than a previous report [30] due to daily media changes to primary colonies to refresh the agonist. These data demonstrate the ability of ERβ activation by a selective agonist to diminish classical stem-cell features of mouse prostate epithelial cells.

### ERβ Agonist Induces Apoptosis in Murine and Human Basal Cells Enriched for Stem Cells

**Figure 3:**
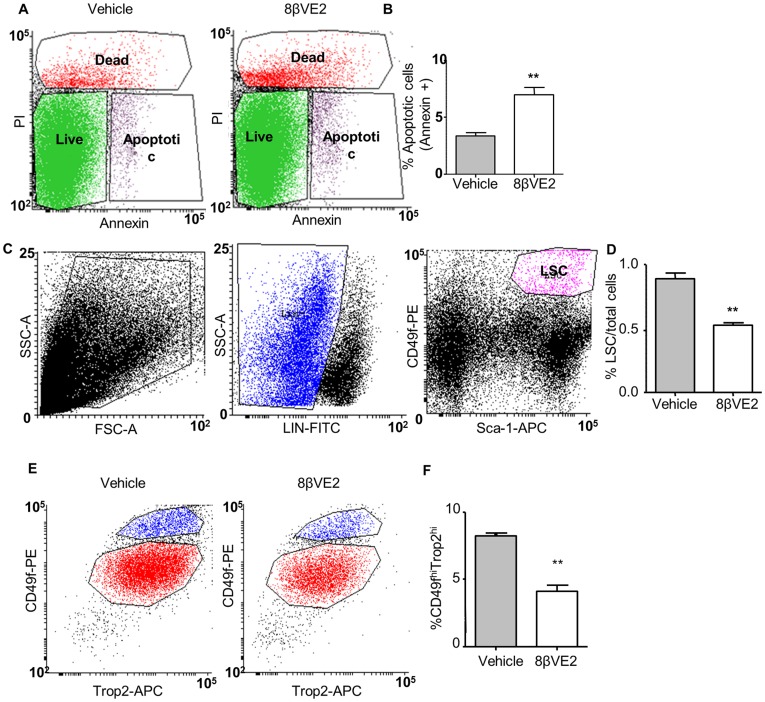
ERβ induces apoptosis in regenerative murine and human prostatic basal cells. (**A**) Representative flow cytometry plots showing live, dead and apoptotic cells following vehicle or 8β-VE2 treatment of digested mouse prostate. (**B**) Percent apoptotic cells following vehicle (grey bar) or 8β-VE2 (open bar) treatment of digested mouse prostate (n = 3 with 3 mice/group/experiment). (**C**) Representative flow cytometry plots showing gating for regenerative murine LSC cells. (**D**) Changes in the regenerative murine LSC population following vehicle (grey bar) or 8β-VE2 treatment (open bar) (n = 3 with 3 mice/group). (**E**) Representative flow cytometry plots following vehicle or 8β-VE2 treatment of human prostatic epithelial cells. (**F**) Changes in the regenerative CD49f^hi^Trop2^hi^ population following vehicle (grey bars) or 8β-VE2 treatment (open bars). (n = 3 patients), **P<0.01 by students t-test.

To further test if 8β-VE2 causes apoptosis in murine enriched stem cells, WT mice were treated for 24 hours with either vehicle or 8β-VE2 and single-cell suspensions of prostate tissue were prepared as previously described [16]. Using flow cytometry, cells were analysed for annexin-V staining to mark for apoptosis from vehicle and 8β-VE2 treated samples (Fig. 3A). Over 3 experiments, 8β-VE2 treatment caused a significant 2.1-fold increase in total levels of cellular apoptosis compared to vehicle controls (Fig. 3B). To test if the stem-enriched population of LSCs (Lin^−^Sca-1^+^CD49f^+^) was directly affected by 8β-VE2, single cell suspensions from the prostate glands of vehicle or agonist treated mice were obtained. Using flow cytometry, the rare LSC population in untreated mice was isolated as previously described [12], [16], [31] (Fig. 3C) and represented ∼1% of the cells. Figure 3D shows a small, but significant decrease (p<0.01) in this fraction of cells after agonist treatment from 0.89±0.09% to 0.53±0.03% (Fig. 3D).

Similarly, the effect of 8β-VE2 on human basal cells that are enriched for stem cells, marked as CD49f^hi^Trop2^hi^, was tested. Following treatment of primary epithelial cells with 8β-VE2 or vehicle for 24 hours, the flow cytometry profiles for CD49f^hi^Trop2^hi^ cells were similar (Fig. 3E), but there was a significant reduction in this population with 8β-VE2 treatment compared to vehicle (p<0.001) (Fig. 3F). Overall these results demonstrate a direct effect of 8β-VE2 on murine and human basal populations containing stem cells.

### ERβ Treatment after Castration Induces a Second Wave of Apoptosis in Castrate-resistant Cells

**Figure 4:**
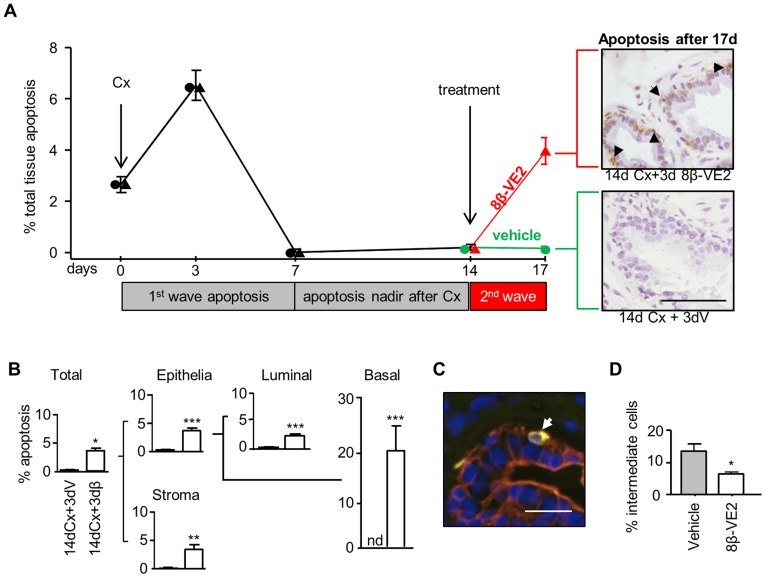
ERβ treatment after castration induces a second wave of apoptosis in castrate-resistant cells, including basal, luminal and intermediate cells. (**A**) Time course of total levels of prostatic apoptosis after 14 days of Cx followed sequentially by 3 days of vehicle (circles) or 8β-VE2 (triangles). Following treatment, groups are coloured green (vehicle) or red (8β-VE2) for clarity (n = 7/group). Representative micrographs show basal cell apoptosis (black arrows indicate apoptag^+^ staining) in 8β-VE2 treated tissues that is absent in vehicle controls. Breakdown of apoptotic levels in individual cell types following sequential treatment (**B–D**). (**B**) Stereological analysis (n≥7/group) showing levels of apoptosis in individual cell types in the prostates of mice treated with 14 day Cx +3 day vehicle (14dCx+3dV) or 14 day Cx +3 day 8β-VE2 (14dCx+3dβ). (**C**) Representative micrograph of immunofluorescent dual labeling in mouse ventral prostate with CKHMW (basal cell marker, green) and CK18 (luminal cell marker, red) used to identify intermediate cells (dual stained in yellow and indicated by white arrow) for quantitation in sequentially treated tissues. (**D**) Relative numbers of intermediate cells following 14 days of Cx and 3 days of vehicle (grey bars) or 8β-VE2 (open bars) (n≥5/group). (nd; non-detectable, *P<0.05, **P<0.01, ***P<0.005 by Mann Whitney test (B) or students t-test (D)). (Scale A = 20 µm, C = 10 µm).

Castration-induced apoptosis peaks 3 days post treatment, then declines to a nadir on day 7. By day 14 no further apoptosis is detectable [32] (Table S1). To further define the differences between ERβ agonist and castration, the combined effect of ERβ agonist treatment to 14-day castrate mice was evaluated (Fig. 4A). When apoptosis was at a nadir on day 14 in the castrate mice, administration of ERβ for three days stimulated a second peak of apoptosis not observed in vehicle treated castrate mice. On day 17, apoptosis was clearly visible in tissue sections by immunohistochemical staining for apoptosis (Fig. 4A) and, when quantified by stereology, was present in ∼4% of the tissue.

Full quantitation of apoptosis per cell type is shown in Figure 4B, where a significant increase in apoptosis occurs in all cell types exposed to ERβ agonist but consistent with this and previous studies [13], [32], [33], there is no further change in castrate tissues. Although the most striking effect is the increase in the basal cells where, 19.32±4.98% apoptosis was measured, significant stimulation of cell death was also detected in the luminal epithelia and stromal cells.

As well as basal cells, recent studies suggest regenerative stem cells are located within the luminal compartment of the prostatic epithelium [34], [35]. Even the intermediate cells, that simultaneously express basal and luminal cytokeratins (5 & 14, 8 & 18, respectively) [36], are postulated to contain stem cells, although their regenerative capacity is untested and unproven. To evaluate any changes to intermediate cells, we identified and quantitated them based on the dual expression of basal (cytokeratin 5/14) and luminal (cytokeratin 8/18) cell markers (Fig. 4C). The sequential treatment of castration followed by ERβ agonist resulted in a 52% (p = 0.02) decrease in intermediate cell number, as compared to vehicle control (Fig. 4D). Collectively, these data demonstrate that unlike castration, 8β-VE2 targets and induce apoptosis within basal and intermediate cells.

## Discussion

The use of estrogens to treat PCa is an effect mediated through pituitary mediated suppression of androgens. However, there are direct, local actions of estrogens on the prostate, mediated through both estrogen receptor subtypes and the development of estrogen selective modulators has distinguished between the adverse effects mediated through ERα and the beneficial ones mediated by ERβ. Whereas ERα is proliferative and pro-tumorigenic, ERβ is anti-proliferative, pro-apoptotic and anti-metastatic [8], [37]. These dual, opposing actions frequently referred to, as the yin and yang of estrogen hormone action in the prostate cannot be considered alone or in isolation from the actions of androgens.

Androgens promote cell proliferation and differentiation [13], [38], [39]. Castration or ADT is effective because it reduces cell proliferation, but as the rate of proliferation in the human prostate gland is relatively low [13], [40], its most important effect is to promote apoptosis and cell death. In this context, although castration and activation of ERβ share similarities, there are important differences. Previously, we demonstrated that whilst ERβ activation of apoptosis was mediated by the extrinsic pathway via TNFα, castration induced apoptosis is via intrinsic signaling [8]. In the current study, we also show that ERβ activation and castration affect different cellular populations, including castrate-resistant stem/progenitor cells necessary for epithelium regeneration. Whereas stimulation of stem cell activity to promote regeneration is a therapeutic strategy for many adult tissues, including heart, liver and kidney, the goal in managing prostate disease is to prevent regeneration, and thus, activation of ERβ may have therapeutic potential.

Both AR and ERβ are mainly expressed in prostatic epithelium, but the stem/progenitor cells that remain after castration are described as AR negative[18], [41]. This study confirms that these cells are responsive to ERβ agonist. Stem/progenitor cells are located in luminal and basal compartments, and even cells of the intermediate phenotype are postulated to be stem/progenitor cells [12], [28], [29], [34], [35], [42], [43], [44]. Two populations of luminal cells with regenerative capability were described in murine studies; Castration Resistant Nkx3.1 expressing cells (CARNs) and those recently described by Liu and colleagues by lineage tracing [34], [35]. We show a small (∼3%), but significant, increase in luminal cell apoptosis in castrate tissues treated with 8β-VE2. These luminal cells may contain CARNs, but as these are rare (0.7% of total epithelial cells) [34] they were insufficient to directly perform stereological analysis and quantitation on fixed tissue specimens by immunofluorescence. Whether or not the luminal cells are those described by Liu *et al* remains to be determined using the PSA-Cre-ER-T2 mice [35]. However, the ability to target castrate-resistant cells regardless of lineage hierarchy remains clinically relevant.

Basal cells are a heterogeneous population, but can be identified by several methods including morphology, nuclear localization and immunohistochemical markers (such as p63^+^ and CKHMW). Morphological evidence of apoptosis and cell death in the basal epithelia after 8β-VE2 treatment correlates with the functional evidence of impaired regeneration of the epithelium and targeting of the regenerative stem/progenitor population that are reduced in number in the atrophic tissue. Testing of murine prostatic cells using the classical stem cell assays of clonogenicity and self-renewal showed a significant inhibition of these functions by 8β-VE2. Our studies also demonstrated an effect of the agonist on the intermediate cells that express both basal and luminal markers [18], [36], although the regenerative capacity of these intermediate cells remain untested.

Further sub-fractionation of basal cells to obtain the stem/progenitor enriched LSC populations, previously identified in mice, also showed them to be responsive to 8β-VE2. Cell death was increased, and the numbers of cells decline in these fractions, consistent with their proven regenerative capacity and the ability of agonist to block regeneration *in vivo*.

Similarly, flow cytometry of human specimens for CD49f^hi^Trop2^hi^ isolates p63+ basal cells, including regenerative stem/progenitor cells, [12], [29] and we showed an 8β-VE2-induced decrease this population. The CD49f^hi^Trop2^hi^ basal cells were also proven to be cells of origin of PCa and therefore demonstrate another potential advantage of 8β-VE2. Altogether, these data are consistent with our previous demonstration that 8β-VE2 induces apoptosis in androgen dependent human prostate cancer cell lines and xenografted primary specimens from men with prostate cancer. Further support for a potential beneficial role for ERβ activation in PCa comes from a recent study by Mak and colleagues, who stimulated ERβ in prostate cancer cells and showed inhibition of epithelial-mesenchymal transition and invasion [37]. Application to other solid tumors such as colon cancer are also predicted, as 8β-VE2 decreased p63 transcript levels in ovariectomized rats [45].

The body of research on androgen signaling in the prostate gland spans several decades and its effective blockade is a proven therapy for prostatic disease. The actions of estrogens on the prostate gland are usually considered independently, with a focus on defining the dual opposing actions through the two receptor subtypes; the beneficial, pro-apoptotic effects via ERβ activation and the adverse effects mediated through ERα [11]. Here we show that combining androgen deprivation and estrogen stimulation with an ERβ specific agonist, are additive because the cellular targets are different. This combination may be strategically advantageous, but it remains imperative to characterize the precise timing of these treatments in the context of disease progression.

Overall, these data provide compelling evidence of the added benefit of using selective agonists of ERβ to enhance the effectiveness of castration and ADT through the regulation of apoptosis in mouse and human specimens. Not only is ERβ action independent of systemic testosterone levels, but its mechanism of action is different to castration and the underlying cellular targets are uniquely different, being the prostatic stem/progenitor cells with regenerative potential and cells of origin of prostate cancer.

## Supporting Information

Figure S1 **ERβ stimulates apoptosis in basal cells but castration does not.** (**A**) Pie chart depicting tissue cellular composition (basal, luminal and stromal compartments) in an untreated/normal prostate. (**B**) Percent apoptosis in luminal cells after 3 days of vehicle (grey bars), castration (Cx, closed bars) or 8β-VE2 treatment (open bars) (n = 5/group). (**C**) Percent apoptosis in basal cells after 3 days of vehicle (grey bars), Cx (closed bars) or 8β-VE2 (open bars) (n = 5/group). (nd; non-detectable *P<0.05, **P<0.01, ***P<0.001, one way ANOVA with Tukeys post-hoc analysis).
(TIF)

Table S1 **Time course of total apoptosis in castrate and ERβ agonist treated mice.** Shown here are total percentages of apoptosis in the ventral prostates of mice following castration or ERβ agonist treatment.
(DOCX)
