# Supplementary material for: Estrogen Receptor β Activation Impairs Prostatic Regeneration by Inducing Apoptosis in Murine and Human Stem/Progenitor Enriched Cell Populations
Source: PLoS One. 2012 Jul 10;7(7):e40732. doi: 10.1371/journal.pone.0040732 (PMC3393688; doi:10.1371/journal.pone.0040732)
Supplement: Table S1 — Time course of total apoptosis in castrate and ERβ agonist treated mice. Shown here are total percentages of apoptosis in the ventral prostates of mice following castration or ERβ agonist treatment. (DOCX) [file pone.0040732.s002.docx]

| **Total % Apoptosis in ventral prostate** | | |
| --- | --- | --- |
| **Days after treatment** | **Cx** | **8β-VE2** |
| 0 | 2.61 ± 0.5 | 2.80 ± 0.5 |
| 3 | 6.53 ± 1.3 | 6.84 ± 1.7 |
| 7 | 0.01 ± 0.3 | 0.11 ± 0.2 |
| 14 | 0.20 ± 0.3 | 0.19 ± 0.3 |
| 17 | 0.20 ± 0.1 | 0.13 ± 0.2 |

**Table S1**
